# Supplementary material for: Microbial-driven preterm labour involves crosstalk between the innate and adaptive immune response
Source: Nat Commun. 2022 Feb 21;13:975. doi: 10.1038/s41467-022-28620-1 (PMC8861006; doi:10.1038/s41467-022-28620-1)
Supplement: Supplementary file 1 — Supplementary Information [file 41467_2022_28620_MOESM1_ESM.pdf]

Supplementary Information file

Title: Microbial-driven preterm labour involves crosstalk between the innate and adaptive immune response

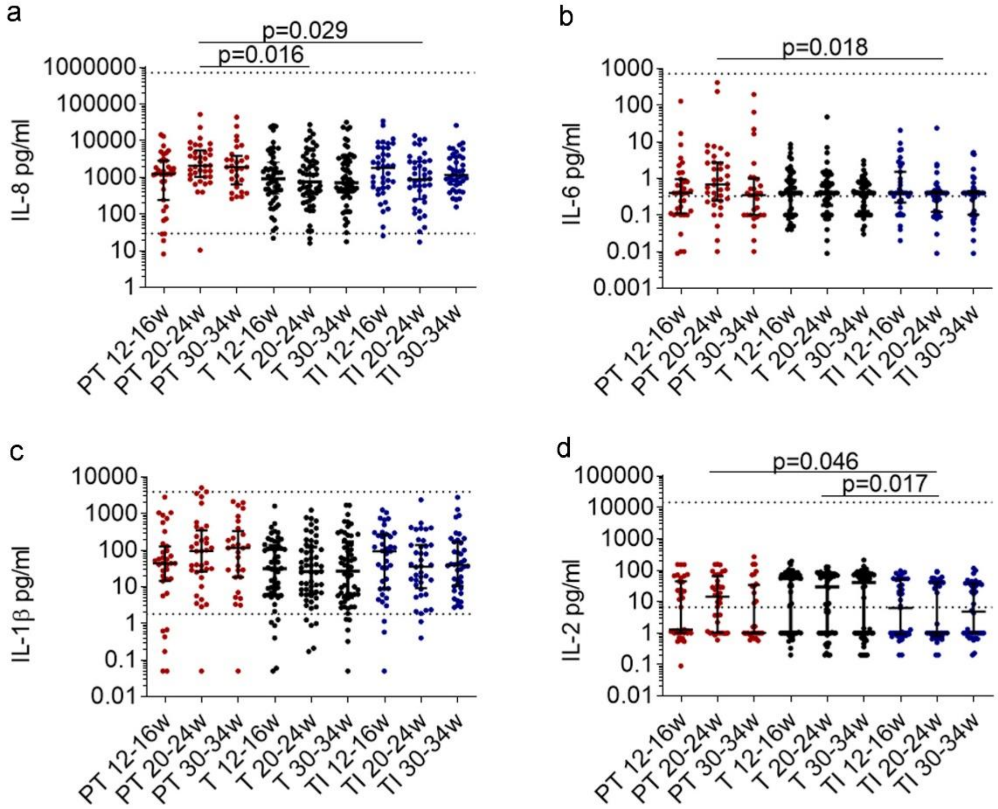

Supplementary figure 1. Cervicovaginal pro-inflammatory cytokines increase in mid pregnancy in women who deliver preterm compared to delivery at term. Cervicovaginal fluid was analysed from 133 women at high risk of preterm birth at three timepoints in pregnancy. 37 women delivered preterm (PT) (providing 35,36 and 29 samples at each timepoint), 56 women delivered at term with no intervention (TU) (providing 52,57 and 57 samples at each timepoint) and 40 women delivered at term following intervention (TI) (providing 39, 40 and 40 samples at each timepoint). Concentrations of IL-8 **a**, IL-6 **b**, IL-1β **c** and IL-2 **d** are shown with median and interquartile ranges. Statistical analyses were performed using the Kruskal Wallis test with Dunn's multiple comparison correction. All samples were biologically independent, with one participant providing two independent samples between 20-24 weeks and one participant providing two independent samples between 30-34 weeks. Data are presented as median values and interquartile ranges (25th and 75th percentiles).

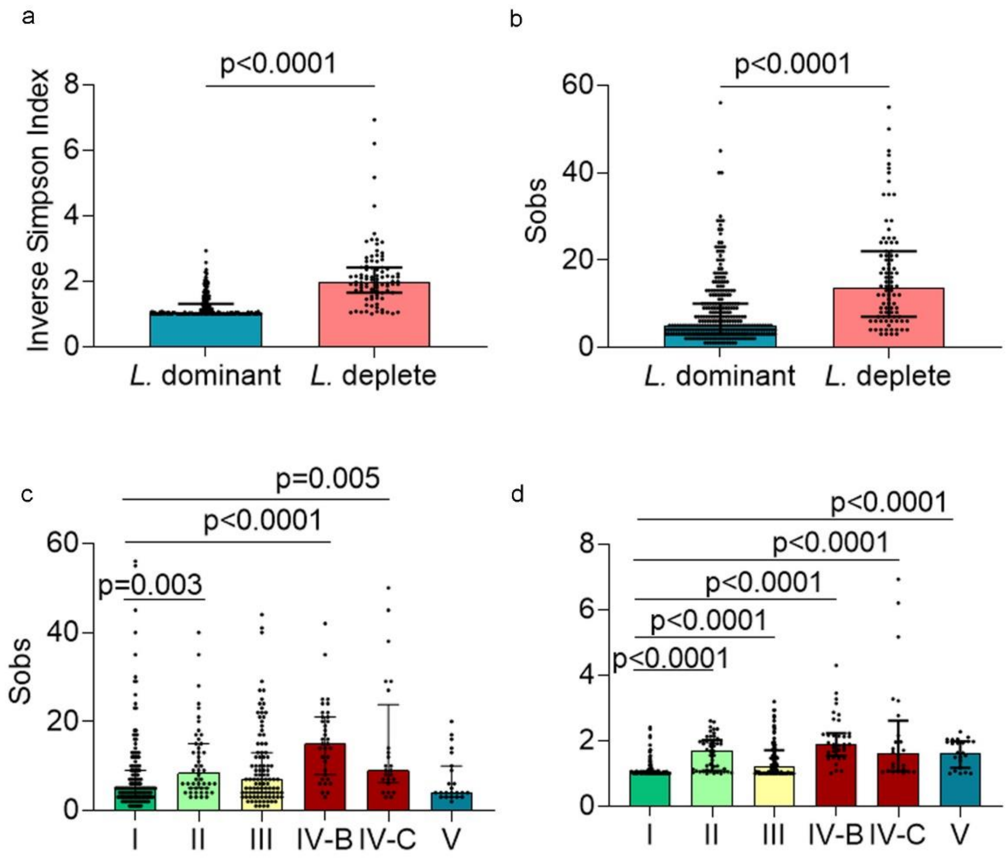

Supplementary figure 2. Species diversity and richness is altered depending on vaginal microbial composition. The Inverse Simpson index was used as a measure of microbial diversity. Microbial diversity **a** and richness **b** were significantly increased with *Lactobacillus* depletion compared to *Lactobacillu*s dominance. A one-sided Mann Whitney was used for statistical analysis. Microbial diversity **c** was significantly increased in CST II (*L. gasseri*), III *(L.iners)*, IV-B (diverse), IV-C (diverse) and V (*L.jensenii*) compared with CST I (*L. crispatus)*, and richness **d** was significantly increased in CST II (*L. gasseri*), , IV-B and IV-C . Kruskal Wallis with Dunn's multiple comparison's test was used for statistical analysis. N=385 samples from n=133 women. Data are presented as median values and interquartile ranges (25th and 75th percentiles).

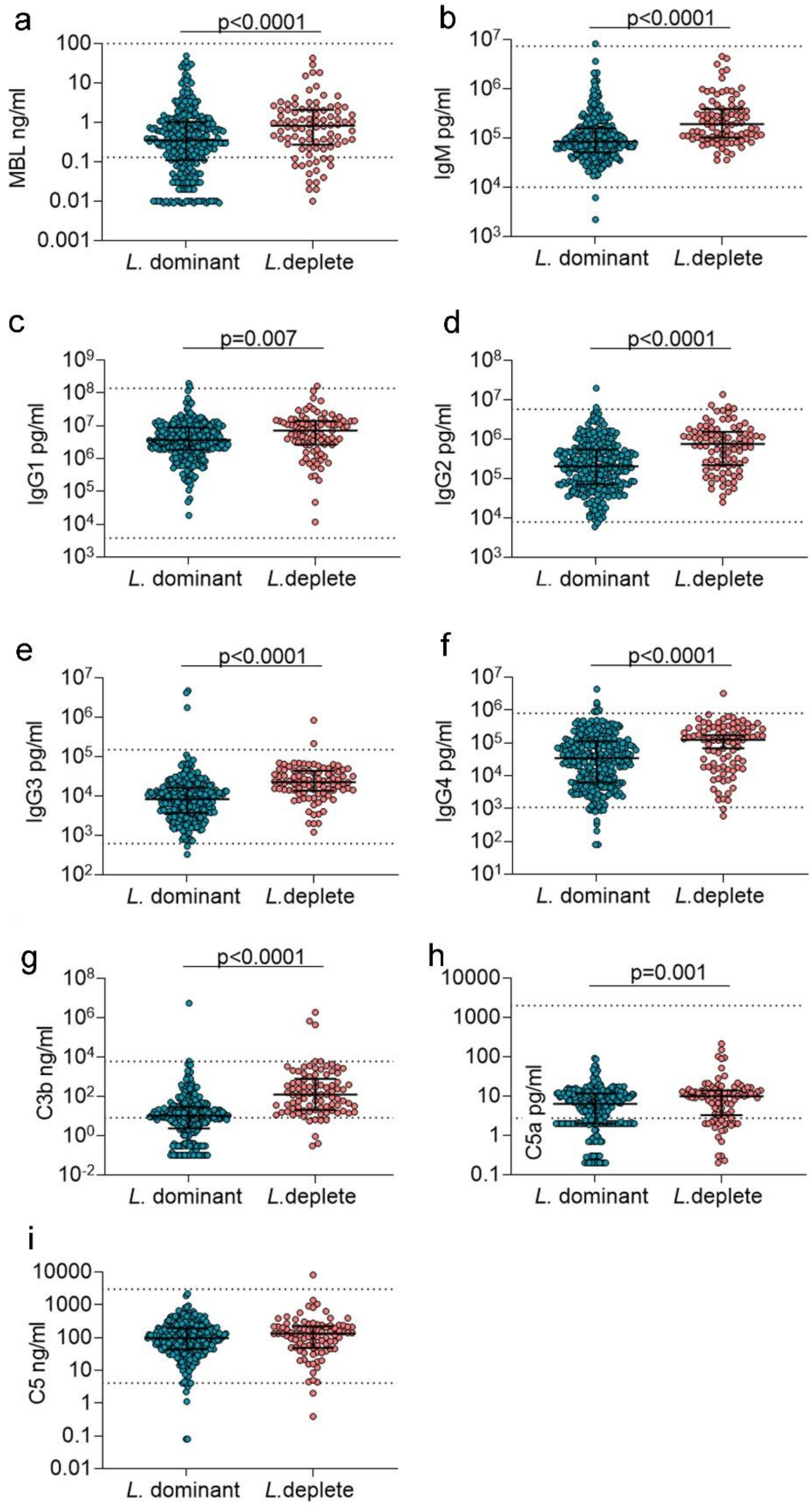

Supplementary figure 3. Cervicovaginal concentrations of mediators of microbial recognition and complement are increased in association with *lactobacillus* depletion. Cervicovaginal concentrations of MBL, IgM, and Ig1-IgG4 were compared between samples taken from women who were *Lactobacillus* dominant and deplete, n=385 samples from n=133 women **a-f**. Statistical analysis was performed using a one-sided Mann Whitney test. Concentrations of C3b, C5 and C5a were compared between samples taken from women who were *Lactobacillus* dominant and deplete **g-i**, n=385 samples from n=133 women. Statistical analysis was performed using a one-sided Mann Whitney test. Data are presented as median values and interquartile ranges (25th and 75th percentiles).

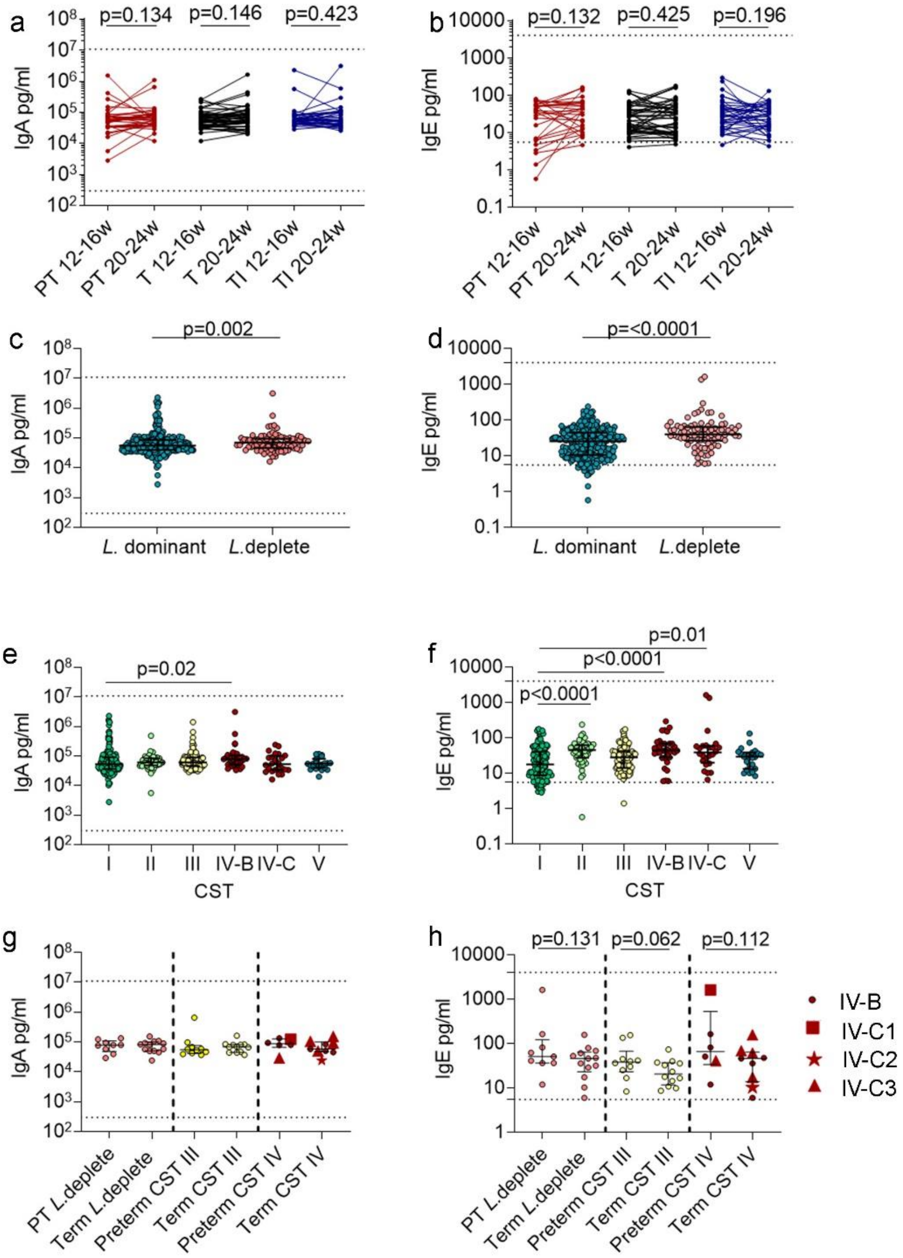

Supplementary figure 4. Cervicovaginal IgA and IgE concentrations. Cervicovaginal IgA and IgE were measured and compared between 12-16 weeks and 20-24 weeks in women who delivered preterm, term without intervention, and at term following intervention **a and b**, n=122. Statistical analysis was performed using a one-sided Wilcoxon matched pairs signed rank test. IgA and IgE were also compared between samples taken from women who were *Lactobacillus* dominant and deplete **c and d**, n=385 samples from n=133 women. Statistical analysis was performed using a one-sided Mann Whitney test. IgA and IgE were compared between samples taken from women who were classed as CST 1-V **e and f**, n=385 samples from n=133 women. The Kruskal Wallis and Dunn's multiple comparison's test was used to determine statistical significance. IgA and IgE was compared in women who delivered preterm compared to at term according to microbial composition at 20-24 weeks **g and h**, statistical analysis of was performed using a one-sided Mann Whitney test with no multiple comparisons adjustments. Data are presented as median values and interquartile ranges (25th and 75th percentiles).

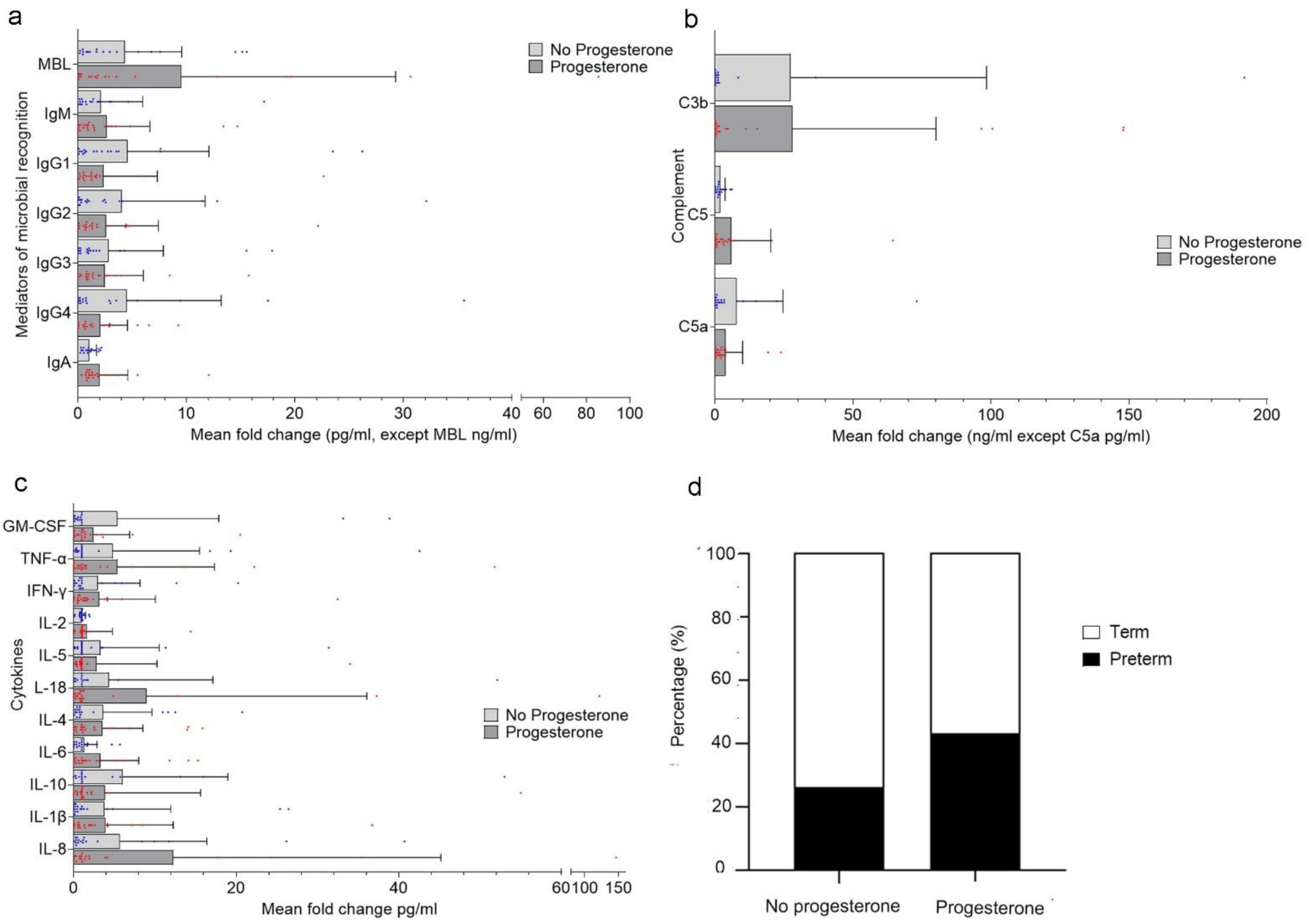

Supplementary figure 5. Progesterone has no effect on the local immune milieu or delivery outcome.
Cervicovaginal immune mediators were analysed from women pre and post cervical cerclage. 20 women received progesterone, and 19 women did not. The fold change concentration of mediators of microbial recognition **a**, complement proteins **b**, and cytokine concentrations **c** between samples pre-cerclage and post-cerclage are compared between women who had progesterone and women who did not (n=20 and n-19 respectively). The percentage of women who delivered preterm in women who did not receive progesterone was compared with women who did receive progesterone **d**. Statistical analysis was performed using one- sided Mann Whitney test and a one-sided Fisher's exact test. Data are presented as mean and standard deviation.

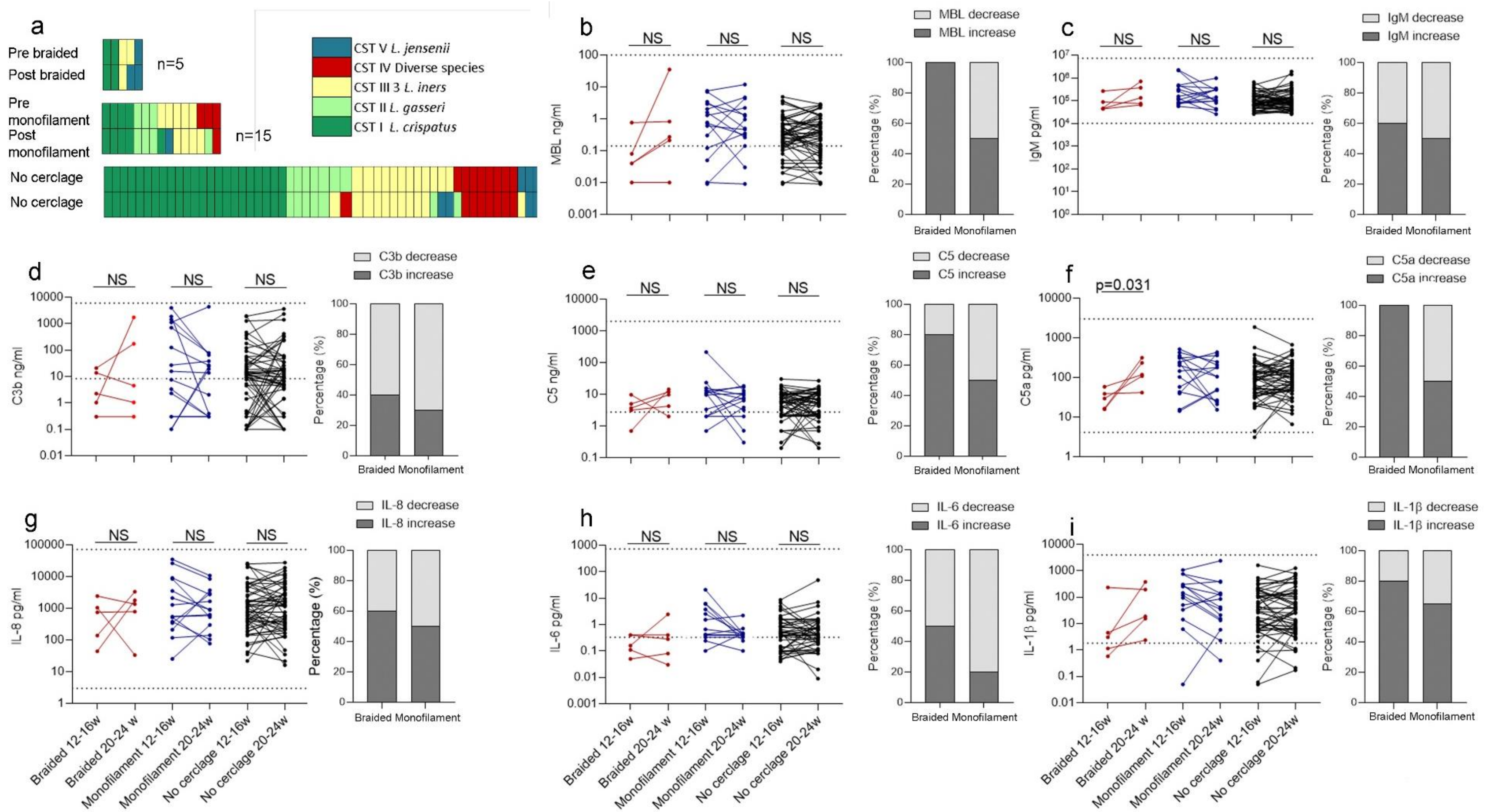

Supplementary figure 6. Braided cervical cerclage material is not associated with activation of complement and cytokine production in women who deliver at term. Vaginal microbiota composition (a) and cervicovaginal immune mediators (**b-i**) were analysed in 73 women who delivered at term between 12-16 weeks and again between 20-24 weeks. 5 women had a cerclage using braided material and 15 women had a cerclage using monofilament material between timepoints, whereas 53 women had no intervention **a**. The concentration of MBL **b**, IgM **c**, C3b **d**, C5 **e**, C5a **f**, IL-8 **g**, IL-6 **h** and IL-1β **I** were compared between 12-16 and 20-24 weeks in women who had a braided cerclage, monofilament cerclage and no cerclage. The proportion of women who had an increase in immune mediators were also compared between suture material. Statistical analysis was performed using one-sided Wilcoxon matched pairs signed rank test and one-sided Fisher's exact test, N=73 women.

Supplementary Table 1. Study population demographics

	Preterm (PT) (<37 weeks)	Term (T) no intervention	Term intervention (TI)	*p* value (PT vs T vs TI)
N (%)	37 (28%)	56 (42%)	40 (30%)	
Gestation at delivery (weeks) median and IQ range	33^{+3} (31^{+5} - 36^{+0})	39^{+5} (39^{+0} - 40^{+5})	38^{+3} (38^{+0} - 39^{+0})	One-way ANOVA p<0.0001 **** PT vs T p<0.0001 **** PT vs TI p<0.0001 ****
Early preterm </=33+6	21 (57%)	NA	NA	
Late preterm >/=34+0	16 (43%)	NA	NA	
Age median and IQ range	31 (28-38)	33 (30-36)	33 (31-36)	One-way ANOVA p=0.93
BMI median and IQ range kg/m^2	24 (22-28)	22 (20-27)	26 (23-28)	One-way ANOVA p=0.20
Ethnicity<br>White N (%)<br>Black N (%)<br>Other N (%)	 18 (48%)<br>9 (24%)<br>10 (28%)	 40 (72%)<br>7 (12%)<br>9 (16%)	 19 (48%)<br>12 (30%)<br>9 (22%)	χ^2 =0.09
Parity<br>Nulliparous<br>Multiparous	 11 (30%)<br>26 (70%)	 31 (55%)<br>25 (45%)	 14 (35%)<br>26 (65%)	
Cervical cerclage	21 (57%)	0	38 (95%)	
History indicated cervical cerclage and material	8/21 (38%) (Mersilene 5, Nylon 3)	NA	24/38 (63%) (Mersilene 12, Nylon 12)	
USS indicated cervical cerclage and material	13/21 (62%) (Mersilene 8, Nylon 4, Unknown 1)	NA	14/38 (37%) (Mersilene 2, Nylon 12)	
Progesterone only	3	NA	2	
Risk factor for PTB: (by individual)				

Cervical treatment	6	34	8	
Cervical treatment + MTL	1	2	3	
Cervical treatment + PTB	7	0	1	
MTL	7	5	13	
MTL + PTB	1	2	6	
PTB	15	12	8	
3x 1st trimester miscarriage	0	1	0	
Short cervix at anomaly	0	0	1	
Risk factor for PTB [(a)]				χ^2 p<0.0001 ****
Cervical treatment	14 (38%)	36 (64%)	12 (30%)	
Previous PTB	23 (62%)	14 (25%)	15 (38%)	
Previous MTL	9 (24%)	9 (16%)	22 (55%)	

BMI=body mass index, USS=ultrasound, PTB=preterm birth, MTL=mid trimester loss, misc=miscarriage. Data presented as median (interquartile range (IQ)) or number (%). P values: One way ANOVA for multiple comparisons or Chi squared for proportional data.

[(a)]Some study participants will have more than one risk factor for PTB.

Supplementary Table 2. Median cervicovaginal cytokine concentrations in women who deliver preterm and at term

Cytokine pg/ml	PT 12-16w	PT 20-24w	p value	T 12-16w	T 20-24 w	p value	TI 12-16w	TI 20-24w	p value
IFN-γ<br>Range:<br>4.7-10290	4.71	5.36	0.29	4.51	4.83	0.69	5.32	3.69	0.05
TNF-α<br>Range:<br>0.94-2050	0.29	0.69	0.33	0.66	0.84	0.81	0.29	0.23	0.74
GM-CSF<br>Range:<br>1.28-2800	0.55	0.81	0.25	0.23	0.39	0.28	0.38	0.27	0.57
IL-18<br>Range:<br>1.73-3790	2.01	2.59	0.31	8.56	9.76	0.98	1.31	1.03	0.83
IL-4<br>Range:<br>1.55-3380	3.78	5.97	0.29	2.06	5.84	0.41	2.92	2.36	0.59
IL-5<br>Range:<br>0.66-1440	0.20	0.25	0.25	1.00	1.00	0.47	0.20	0.20	0.98

PT preterm, T term, TI term with intervention. Range: standard curve range. Statistical analysis was performed using a one-sided Wilcoxon matched pairs signed rank test. PT 12-16 weeks N=35, PT 20-24 weeks N=36, PT 30-34 weeks N=29, T 12-16 weeks N=52, T 20-24 weeks N=57, T 30-34 weeks N=57, TI 12-16 weeks N=39, TI 20-24 weeks N=40, TI 30-34 week N=40.

Supplementary Table 3. Median cervicovaginal cytokine concentrations and Lactobacillus dominant and deplete samples

Cytokine pg/ml	IFN-γ Range: 4.7-10290	TNF-α Range: 0.94-2050	GM-CSF Range: 1.28-2800	IL-18 Range: 1.73-3790	IL-4 Range: 1.55-3380	IL-5 Range: 0.66-1440
Lactobacillus dominant	4.53	0.31	0.37	2.02	3.03	1.00
Lactobacillus deplete	3.43	0.69	0.26	1.86	3.74	1.00
P value	0.15	0.11	0.12	0.36	0.09	0.24

Cytokines were compared between samples taken from women who were *Lactobacillus* dominant and deplete, n=385 samples from n=133 women. Statistical analysis was performed using a one-sided Mann Whitney test.

Supplementary Table 4 Catalogue numbers for immunoassays

Immunoassay kit	Company	Catalogue number
IL-8	R&D Systems/Bio-Techne	hMagnetic Luminex Assay 1-Plex
10-plex cytokines (IL-1β, IL-2, IL-4, IL-5, IL-6, IL-10, IL-18, IFN-γ, GM-CSF and TNF-α)	R&D Systems/Bio-Techne	hMagnetic Luminex Assay 10-Plex
MBL,C5 C5a	Merck/ Millipore	HCMP1MAG-19K-03
C3b/iC3b	Merck/Millipore	HCMP2MAG-19K-01
7-plex Immunoglobulins (IgM, IgG1, IgG2, IgG3, IgG4, IgA and IgE)	Thermofisher	
