# Supplementary material for: Microbial-driven preterm labour involves crosstalk between the innate and adaptive immune response
Source: Nat Commun. 2022 Feb 21;13:975. doi: 10.1038/s41467-022-28620-1 (PMC8861006; doi:10.1038/s41467-022-28620-1)
Supplement: Supplementary file 3 — Description of Additional Supplementary Files [file 41467_2022_28620_MOESM3_ESM.pdf]

Description of Additional Supplementary Files

File Name: Supplementary Data 1
Description: Table of study metadata, microbial composition, and ENA accession numbers.

File Name: Supplementary Data 2
Description: Table presenting microbial composition by study sample.
