# Supplementary material for: Microbial-driven preterm labour involves crosstalk between the innate and adaptive immune response
Source: Nat Commun. 2022 Feb 21;13:975. doi: 10.1038/s41467-022-28620-1 (PMC8861006; doi:10.1038/s41467-022-28620-1)
Supplement: Supplementary file 6 — Reporting Summary [file 41467_2022_28620_MOESM6_ESM.pdf]

nature research

Corresponding author(s): Dr Lynne Sykes

Last updated by author(s): Dec 21, 2021

# Reporting Summary

Nature Research wishes to improve the reproducibility of the work that we publish. This form provides structure for consistency and transparency in reporting. For further information on Nature Research policies, see our Editorial Policies and the Editorial Policy Checklist.

## Statistics

For all statistical analyses, confirm that the following items are present in the figure legend, table legend, main text, or Methods section.

| n/a | Confirmed | |
|---|---|---|
| ☐ | ☒ | The exact sample size ($n$) for each experimental group/condition, given as a discrete number and unit of measurement |
| ☐ | ☒ | A statement on whether measurements were taken from distinct samples or whether the same sample was measured repeatedly |
| ☐ | ☒ | The statistical test(s) used AND whether they are one- or two-sided<br>*Only common tests should be described solely by name; describe more complex techniques in the Methods section.* |
| ☐ | ☒ | A description of all covariates tested |
| ☐ | ☒ | A description of any assumptions or corrections, such as tests of normality and adjustment for multiple comparisons |
| ☐ | ☒ | A full description of the statistical parameters including central tendency (e.g. means) or other basic estimates (e.g. regression coefficient) AND variation (e.g. standard deviation) or associated estimates of uncertainty (e.g. confidence intervals) |
| ☐ | ☒ | For null hypothesis testing, the test statistic (e.g. $F$, $t$, $r$) with confidence intervals, effect sizes, degrees of freedom and $P$ value noted<br>*Give P values as exact values whenever suitable.* |
| ☒ | ☐ | For Bayesian analysis, information on the choice of priors and Markov chain Monte Carlo settings |
| ☒ | ☐ | For hierarchical and complex designs, identification of the appropriate level for tests and full reporting of outcomes |
| ☐ | ☒ | Estimates of effect sizes (e.g. Cohen's $d$, Pearson's $r$), indicating how they were calculated |

*Our web collection on statistics for biologists contains articles on many of the points above.*

## Software and code

Policy information about availability of computer code

| | |
|---|---|
| Data collection | No software was used for data collection. |
| Data analysis | Data analysis was performed on Graphpad Prism version 9.0.0 and stored on Graphpad files. The R package v3.6 was also used. FastQC v1.6, Qiime2 pipeline v 2021.4 and DADA2v3.14 were used for microbiome analyses. |

For manuscripts utilizing custom algorithms or software that are central to the research but not yet described in published literature, software must be made available to editors and reviewers. We strongly encourage code deposition in a community repository (e.g. GitHub). See the Nature Research guidelines for submitting code & software for further information.

## Data

Policy information about availability of data

All manuscripts must include a data availability statement. This statement should provide the following information, where applicable:

- Accession codes, unique identifiers, or web links for publicly available datasets
- A list of figures that have associated raw data
- A description of any restrictions on data availability

The data sets generated during and/or analysed during the current study are available as Data Source Files 1 and 2. Graphs from figures were constructed from GraphPad data files, with raw data contained in excel spreadsheets in Data Source files 1 and 2. All sequence data is available on the ENA database browser https://www.ebi.ac.uk/ena, with accession codes linked to sample type within Data Source file 1.
The data availability statement reads as follows following recommendation by the author guidances checklist document:"Source data are provided with this paper. Data Source File 1 includes metadata including ENA accession numbers, Data Source File 2 includes the raw data used to create each figure. All sequence data is available on the ENA database browser https://www.ebi.ac.uk/ena. "

# Field-specific reporting

Please select the one below that is the best fit for your research. If you are not sure, read the appropriate sections before making your selection.

☒ Life sciences ☐ Behavioural & social sciences ☐ Ecological, evolutionary & environmental sciences

For a reference copy of the document with all sections, see nature.com/documents/nr-reporting-summary-flat.pdf

# Life sciences study design

All studies must disclose on these points even when the disclosure is negative.

| | |
|---|---|
| Sample size | Study participants were prospectively recruited from preterm birth prevention clinics from five UK hospitals (Chelsea Westminster Hospital, Edinburgh Royal Infirmary, St Marys Hospital London, Queen Charlottes Hospital, and University College London Hospital) between February 2016 and June 2018. A total of 133 women were recruited from these clinics, providing a cohort enriched with women at high risk of preterm birth, with a preterm birth rate of 27.82% (almost three times the national average). There were no power calculations as there was no information in the literature on the effect of the microbiome on complement and adaptive immune response in pregnancy /preterm birth to help ascertain a power calculation. We therefore selected a study time frame and the cohort n number was determined this way. The rationale to support sufficient sample size is since we saw significant effects with this sample size. |
| Data exclusions | There were no data exclusions. |
| Replication | No replication cohorts were included in the study because no replication cohorts were tested. Reproducibility of luminex immunoassays were performed with validation controls to ensure no plate to plate variation or batch effects existed. Validation controls included the standard curves and internal controls that were used from plate to plate to demonstrate no plate to plate variation or batch effect. All analytes were tested to ensure this reproducibility. |
| Randomization | All pregnant women who were eligible for the study were approached to take part, therefore there was no randomization. Outcomes were known following delivery, which was how the groups were identified retrospectively, i.e. preterm delivery and term delivery. |
| Blinding | Samples were analysed with delivery outcome known by clinician and researcher therefore blinding to outcome was not possible. |

# Reporting for specific materials, systems and methods

We require information from authors about some types of materials, experimental systems and methods used in many studies. Here, indicate whether each material, system or method listed is relevant to your study. If you are not sure if a list item applies to your research, read the appropriate section before selecting a response.

## Materials & experimental systems

| n/a | Involved in the study | |
|---|---|---|
| ☒ | ☐ | Antibodies |
| ☒ | ☐ | Eukaryotic cell lines |
| ☒ | ☐ | Palaeontology and archaeology |
| ☒ | ☐ | Animals and other organisms |
| ☐ | ☒ | Human research participants |
| ☒ | ☐ | Clinical data |
| ☒ | ☐ | Dual use research of concern |

## Methods

| n/a | Involved in the study | |
|---|---|---|
| ☒ | ☐ | ChIP-seq |
| ☒ | ☐ | Flow cytometry |
| ☒ | ☐ | MRI-based neuroimaging |

## Human research participants

Policy information about studies involving human research participants

| | |
|---|---|
| Population characteristics | Study participants were prospectively recruited from preterm birth prevention clinics from five UK hospitals. Women were at high risk of preterm birth, either due to a history of previous sPTB, previous mid-trimester loss, recurrent miscarriage, incidental finding of cervical shortening and/or cervical excisional treatment. Samples were collected at three timepoints in pregnancy -timepoint A (12+0 - 16+6 weeks) and at two further study timepoints B (20+0 - 24+6 weeks) and C (30+0 - 34+6 weeks) unless delivery occurred before. Exclusion criteria were women under the age of 18, HIV or hepatitis C positive status, and vaginal intercourse or bleeding within 72 hours of sample collection. Covariate relevant characteristics of research participants include ethnicity, parity, and risk factor for preterm birth and are stated in table 1 of the supplementary information file. |
| Recruitment | Women were provided with information on the study, and were recruited by clinical research staff in the clinic setting. Written consent was obtained. All eligible women were approached in order to prevent selection bias. |

| | |
|---|---|
| Ethics oversight | The study was approved by the National Health Service, National Research Ethics Committee in Stanmore, London (REC 14/LO/0328) |

Note that full information on the approval of the study protocol must also be provided in the manuscript.
